# Clinical Outcomes and Management in Late Diagnosed Siblings Affected With Attenuated GSD Ib

**DOI:** 10.1002/jmd2.70079

**Published:** 2026-02-26

**Authors:** Gregory Lynch, Alison Woodall, Charlotte Dawson, Philip Newsome, Maria Veiga‐da‐Cunha, Karolina M. Stepien

**Affiliations:** ^1^ Clinical Biochemistry Department Northern Care Alliance NHS Foundation Trust Salford UK; ^2^ Adult Inherited Metabolic Diseases Northern Care Alliance NHS Foundation Trust Salford UK; ^3^ Adult Inherited Metabolic Disorders University Hospitals Birmingham NHS Foundation Trust Birmingham UK; ^4^ Faculty of Life Sciences and Medicine, King's College London, Foundation for Liver Research & King's College Hospital Roger Williams Institute of Liver Studies London UK; ^5^ Groupe de Recherches Metaboliques, de Duve Institute UCLouvain Brussels Belgium; ^6^ Division of Cardiovascular Sciences University of Manchester Manchester UK

**Keywords:** attenuated, empagliflozin, glycogen storage disease Ib, long‐term outcome, natural history

## Abstract

Glycogen storage disease 1b (GSD1b) typically presents in early infancy with poor fasting tolerance, hepatomegaly, and neutropenia. We report two siblings who were diagnosed with GSD1b in adulthood. Both had a normal fasting tolerance throughout childhood and, as adults, were able to fast for at least 16 h without developing hypoglycaemia. The older sibling developed nodular cirrhosis during adolescence. The younger sibling exhibited a more pronounced metabolic phenotype, including hyperuricaemia leading to recurrent gout and nephrolithiasis. He experienced occasional episodes of mild neutropenia that were corrected with empagliflozin treatment. To our knowledge, these represent the first reported patients with GSD1b presenting in adulthood with non‐hypoglycaemic complications of the disease and without overt neutropenia or neutrophil dysfunction.

## Highlights

- GSD1b usually presents in early childhood with hypoglycaemia, lactic acidosis, hepatomegaly and neutropenia.
- GSD1b patients can rarely withstand prolonged fasting periods without hypoglycaemic symptoms. The absence of these symptoms contributed to the late diagnosis in these two patients.
- Differing phenotypes and severity can be present even in the same underlying GSD1b genotype.

## 1 Introduction

**FIGURE 1:**
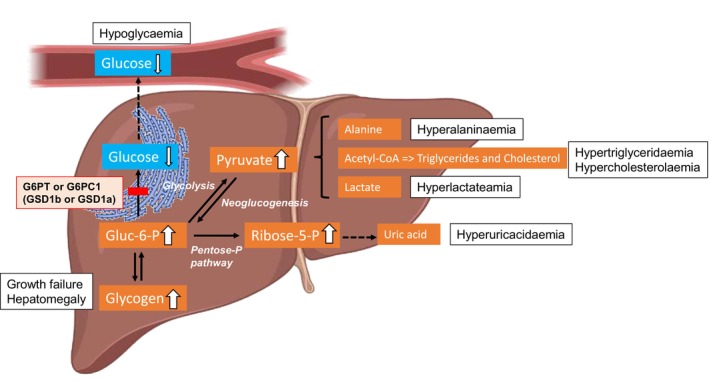
Consequences of deficiencies in glucose‐6‐phosphate translocase (G6PT) in GSD1b or glucose‐6‐phosphatase (G6PC1) in GSD1a on liver metabolism. The inability to dephosphorylate glucose‐6‐phosphate (Glc‐6‐P) leads to its accumulation in hepatocytes, resulting in increased pyruvate production through glycolysis and enhanced lipogenesis, as well as elevated alanine and lactate synthesis. Because Glc‐6‐P is also diverted into the pentose phosphate pathway, ribose‐5‐phosphate levels rise, partially contributing to elevated uric acid production. This effect is further amplified by increased ATP catabolism and subsequent depletion, triggered by hypoglycaemia and the breakdown of nucleotides into uric acid.

Glycogen storage disease 1b (GSD1b) (OMIM #2322) is an autosomal recessive metabolic disorder caused by pathogenic variants in the SLC37A4 gene (OMIM#232220), located on chromosome 11q23.3. The genetic defect inactivates the glucose‐6‐phosphate transporter (G6PT) of the endoplasmic reticulum (ER). This impairs transport into the endoplasmic reticulum of (1) glucose‐6‐phosphate leading to excessive hepatic and renal glycogen accumulation as well as (2) 1,5‐anhydroglucitol‐6‐phosphate (1,5‐AG6P), which underlies the neutropenia and neutrophil dysfunction characteristic of GSD1b [1, 2]. In liver, excess intracellular glucose‐6‐phosphate, which cannot be dephosphorylated, is instead metabolized to pyruvate and lactate, and diverted to lipogenesis and to the pentose phosphate pathway. This contributes to the lactic acidosis, the hypertriglyceridemia and the rise in serum uric acid characteristic of both GSD1b and GSD1a [3]. The overall pathological cascade is displayed in Figure 1.

GSD1b usually presents as fasting hypoglycaemia and hepatomegaly in childhood before the age of 6 months [4, 5]. Only rare, isolated patients with GSD1b [6] or the related condition GSD Ia [7, 8, 9, 10, 11], with late diagnosis and milder pathogenic variants have been described in the literature.

Patients with GSD1b typically have persistent intolerance to fasting into adulthood although fasting tolerance gradually improves with age. Consequently, the mainstay of treatment for GSD1b is dietary modification to maintain normoglycaemia with regular snacks and meals supplemented with cornstarch (or other complex sugars) as a long‐acting source of glucose [12]. Whilst the length of fasting tolerance increases during adulthood, patients remain at risk of acute metabolic decompensation from hypoglycaemia and metabolic acidosis [13]. Progressive liver disease from cirrhosis, hepatic adenomas, and hepatocellular carcinoma (HCC) are a major cause of morbidity. In one case series, 8% of patients with GSD1b developed hepatocellular carcinoma with a mean age of diagnosis of 26 years and an average of 7 years from adenoma detection to development of HCC [14]. In severe cases associated with multiple adenomas and/or hepatocellular carcinoma or decompensated cirrhosis, orthotopic liver transplantation transforms prognosis by reversing the risk of death from hepatic complications and correcting hypoglycaemia. However, the extrahepatic manifestations of neutropenia persist [15, 16].

Historically, neutropenia was managed with granulocyte colony‐stimulating factor (G‐CSF) to stimulate neutrophil production in the bone marrow. Yet, with G‐CSF therapy, patients continue to accumulate 1,5‐AG6P, explaining why this only marginally helps to alleviate the immunological manifestations associated with low neutrophil counts [17].

Specific clinical features of GSD1b include neutropenia and neutrophil dysfunction, which predispose patients with GSD1b to recurrent ulcerations and infections such as gingivitis, oral ulcers, respiratory tract infections, abscesses, and enterocolitis [18]. The underlying cause was recently linked to the accumulation of 1,5‐AG6P in neutrophils, a potent inhibitor of hexokinases [2]. As a result, neutropenia in GSD1b is now often managed with a novel therapeutic approach that directly targets the toxic build‐up of 1,5‐AG6P in neutrophils [19, 20, 21]. This strategy repurposes SGLT2 inhibitors, most commonly empagliflozin, originally developed for type 2 diabetes mellitus [16]. By blocking renal glucose reabsorption, these agents indirectly inhibit SGLT5, the renal transporter of 1,5‐AG [22]. The resulting decrease in blood 1,5‐AG levels, the precursor of 1,5‐AG6P, improves neutropenia and neutrophil function [19]. Clinically, this treatment reduces infection frequency and lowers the risk of developing or worsening inflammatory bowel disease [23]. It has also been shown to improve quality of life, diminish or eliminate the need for G‐CSF injections, and reduce healthcare‐associated costs [24, 25]. The focus of this case series is to describe the diagnostic pathway in adulthood, of two siblings presenting with attenuated symptoms in their adolescence, their long‐term management, and clinical outcomes.

## 2 Patient 1

We present a case of a British‐Pakistani male born to consanguineous parents, with normal developmental milestones and initially no health concerns. At the age of 6 months, he was noted to have hepatomegaly and liver function tests showed a slightly raised ALT at 54 IU/L (RR: 7–40). The remaining liver function tests were normal. He had normal development and there were no concerns regarding his growth. At age 17, liver ultrasound scan showed markedly bright and diffusely coarse nodular liver with at least three hypoechoic solid lesions. Further metabolic investigations including plasma amino acids, urine organic acids, plasma lactate, plasma ammonia and acylcarnitine profile at that time did not reveal a cause. He remained on long‐term MRI surveillance under hepatology which over time showed stable appearance of hepatic cirrhotic nodules on a background of steatohepatitis and moderate fibrosis. HisMRI at age 33 (Figure S1) showed cirrhotic liver with multiple siderotic nodules and no suspicious focal hepatic lesions suggestive of HCC. There was persistent mild elevation in serum transaminases with normal synthetic function (Table S1) and undetectable alpha‐fetoprotein (AFP). Upper gastrointestinal endoscopy at the age of 31 showed mild gastritis with no oesophageal or gastric varices. Eighteen months later his AFP was raised to 145 U/L and a repeat MRI scan confirmed an HCC of 11 mm and several dysplastic nodules. He is currently being considered for liver transplantation.

Throughout life, he has been able to fast for prolonged periods, up to 16 h as an adult without obvious symptoms and remains in good general health. He had one episode of gout at 32 years for which he takes regular allopurinol 300 mg once daily.

At the age of 29, he was enrolled in the 100 000 genomes project [26] which identified a homozygous pathogenic variant c.1243C>T p. (Arg415Ter) in the SLC37A4 gene coding for G6PT, consistent with GSD1b.

Neutrophil counts (Table S1) and serum urate were within the normal range on serial measurements. No significant recurrent infections or inflammatory bowel disease symptoms were noted. Interestingly, he was consistently hypocholesterolaemic with only intermittently mildly raised triglycerides. Altogether, this suggested a very mild metabolic phenotype. Blood 1,5‐AG was measured and shown to be in the average range of 150 μmol/L (Table 1) as found in healthy individuals [27], but remarkably he had no history of infections and kept a consistently normal neutrophil count (see Table 1).

**TABLE 1:** Neutrophil counts and concentration of blood 1,5‐AG measured in dry blood spots [28].

Subject	Patient 1 (aged 33)		Patient 2 (aged 29)	
Sample	Baseline	At 3‐month follow‐up	Baseline	At 3‐month follow‐up
Treatment	None	None	None	Empagliflozin (0.17 mg/kg/day)
Blood 1,5‐AG (μM)	142	153	143	**21**
Neutrophil counts (ANC × 10^9^/L) (RR 1.8–7.5)	2.28 (SD ±0.3)	2.4	2.21 (SD ±0.97)	2.8

*Note:* Empagliflozin treatment was started at the dose of 10 mg once daily (0.17 mg/kg/day) and continued at the same dose for 27 months. 1,5‐AG: 1,5‐anhydroglucitol; ANC: absolute neutrophil count; RR‐ reference range.

Concerning his diet, he did not drink alcohol and remarkably was able to fast for religious purposes often between 12 to 18 h without any obvious symptoms. During religious fasting, some advice was given to increase protein intake in a high carbohydrate meal with the meal at night. Continuous glucose monitor (CGM) Libre showed blood glucose between 4.0 to 10.0 mmol/L for 96% of the time. CGM was recorded for one week (age 29) using a Dexcom 6 device and again (age 33 and 34) using an Abbot Libre device (Table S2). The data collected was timed to cover both fasting and non‐fasting days. These measurements did show that blood glucose could drop to between 3.0 and 3.9 mmol/L (normal range was defined at between 4.0 and 10.0 mmol/L) and this was more likely to occur when fasting for prolonged period, i.e., over 10 h. CGM done at the age of 29 years showed that blood glucose did not drop below 4.0 mmol/L. At the age of 33 years, blood glucose was above 10.0 mmol/L for 1% of the time and at 34 years, blood glucose was above 10.0 mmol/L 4% of the time. Plasma lactate values during CGM monitoring is not available. Plasma lactate has never been raised when measured routinely in clinic.

Body weight remained stable during the last 6 years of follow‐up. Mean weight was 70.26 ± 1.06 kg and calorie intake was between 1600 and 2000 kcal daily. Dietary protein remained close to 75 g daily except during fasting, when it was reduced to 60 g daily. His carbohydrate intake accounts for 44% of his total calorie intake.

## 3 Patient 2

A 28‐year‐old male, the younger brother of Patient 1, was born at term and had normal development. Throughout childhood there were no concerns regarding his development and growth (current height 169 cm). In childhood, his liver function tests showed persistently mildly elevated ALT/AST with maximum reported value of 80/69 IU/L, respectively. Clinical examination showed mild hepatomegaly with liver ultrasounds at the age of 13 and 16 years confirming fatty liver. A fibroscan at the same time was normal (3.4–3.5 kilopascals), ruling out fibrosis. A liver MRI at 24 years old showed less pronounced hepatomegaly with less prominent steatohepatitis and nodular change compared with his brother. Like his brother, initial investigations for an underlying aetiology were not forthcoming. He was found to be alpha‐1‐antitrypsin phenotype PM (poor metaboliser) with a low alpha‐1‐antitrypsin level of 0.7 but this was not felt to be a major contributor to his liver disease (Table S3).

At the age of 25, following his brother's diagnosis of GSD1b, he was tested and found to also carry the same homozygous variant c.1243C>T p.(Arg415Ter) in the *SLC37A4* gene.

In adulthood, he developed uric acid with kidney stones appearing around 20 years old and this is now followed up in the urology department. He also had one gout episode and is since treated with allopurinol (300 mg once daily). His usual fasting tolerance is up to 16 h, like his brother. However, subjectively at the age of 25, he reported hypoglycaemia symptoms and minor hypoglycaemic periods were recorded overnight with CGM (glucose 3–3.9 mmol/L). He also showed evidence of osteopaenia on DEXA (Table S4) and was started on vitamin D 1000 IU daily.

No significant infections requiring hospital admission were noted but he recalled experiencing more childhood infections and taking longer to recover than his sibling. Occasionally, he has also suffered from mouth ulcers, and mild neutropenia was also recorded.

We were surprised by the observation that neither of the siblings had a pronounced neutropenia despite their GSD1b diagnosis (Tables 1, S1 and S3). This led to the measurement of their blood concentration of 1,5‐AG (Table 1), in case this would be particularly low, due for example to a heterozygous variant in SGLT5 that lowered renal 1,5‐AG reabsorption. These variants are present in the heterozygous state in approximately 2% of the population [22] and have already been shown to lower blood 1,5‐AG and attenuate neutropenia in a G6PC3‐deficient patient [29]. However, as mentioned above (Table 1), both siblings had blood levels of 1,5‐AG in the average range of 150 μmol/L [27], which did not explain their fairly normal neutrophil counts.

Yet, since sibling 2 had a mild neutropenia (Tables 1 and S3), with neutrophil counts that were, however, well above what is normally seen in other GSD1b patients, and since he showed painful mouth ulceration, it was decided to begin empagliflozin treatment with 10 mg once daily (0.17 mg/kg/day) to try to improve this. This lowered blood 1,5‐AG, corrected the neutropenia (Table 1), and resulted in the remission of the mouth ulcers. Interestingly, the blood 1,5‐AG levels on empagliflozin were about half (±20 μM) of what is commonly seen in empagliflozin‐treated GSD1b adults, likely due to the absence of cornstarch intake [30] (Table 1).

At the age of 28, his CGM readings prior to treatment indicated that he was within the target range of 4.0–10.0 mmol/L for 72% of the recording with the remaining 28% of readings falling in the mildly hypoglycaemic range of 3.0–3.9 mmol/L. This was mainly at night whilst asymptomatic and after not eating prior to sleep. Plasma lactate was marginally raised at 3.21–4.40 mmol/L (0.5–2.2). After treatment with empagliflozin his readings are in target range for 99% of the time with only one episode of hypoglycaemia noted that was very brief and self‐resolved (Table S5). He does not follow a typical GSD1b diet in that he does not take cornstarch, modified starch or have an overnight enteral feed. He is aware of dietetic advice regarding complex carbohydrates. His total carbohydrate intake is approximately 48% of his total calorie intake.

He remained relatively well on limited dietary modification such as regular carbohydrate meals during the daytime. He has not suffered from a further gout attack since starting allopurinol therapy and maintains a normal neutrophil count on empagliflozin. Hepatic surveillance continues to show stable mild steatohepatosis, with normal liver transaminases, synthetic function and undetectable AFP. He has regular liver MRI on an annual basis.

## 4 Discussion

This paper describes the diagnosis and clinical course of two siblings with GSD1b diagnosed in adulthood. Both presented with steatosis and mildly raised serum ALT in childhood, but the older sibling (Patient 1) showed a more marked hepatic pathology with cirrhotic nodular change evident on imaging from adolescence.

**FIGURE 2:**
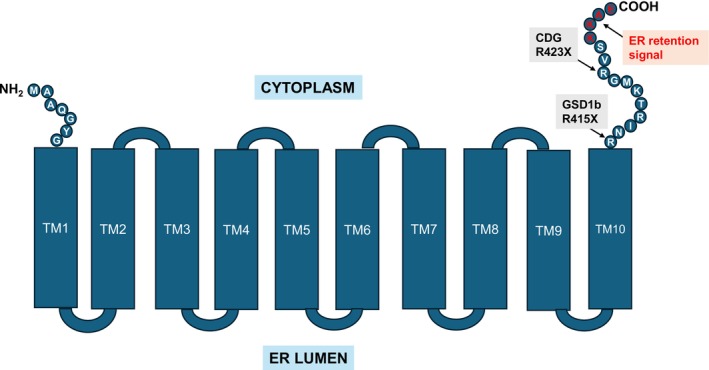
Illustration of the glucose‐6‐phosphate transporter (SLC37A4) showing pathogenic variants R415X (Arg415Ter) mutated in GSD1b and R423X (Arg423Ter) mutated in CDG patients, both located close to the endoplasmic reticulum (ER) retention signal.

The variant found in both siblings, c.1243C>T p.(Arg415Ter) results in the loss of the entire 15 amino acid cytoplasmic tail of the carboxyl‐domain, including the ER retention signal of G6PT (Figure 2 and [1]). Interestingly, the two siblings exhibited a pronounced liver phenotype that partially resembled the liver dysfunction seen in the dominantly inherited congenital disorder of glycosylation caused by a *de novo* variant in SLC37A4 c.1267C>T (p.Arg423Ter) [29, 31, 32], rather than the typical GSD1b presentation characterized by hypoglycaemia and neutropenia. This atypical presentation delayed diagnosis until late adulthood. Consequently, we decided to analyse transferrin glycosylation, which was found to be normal, thereby excluding glycosylation defects as the cause of the observed liver dysfunction (Table S3), as was seen in the patients with the congenital disorder of glycosylation.

The Arg415Ter variant has previously been reported in the literature, both in a compound heterozygous state, in a GSD1b patient without obvious signs or symptoms of neutropenia [33], and in two siblings of Afro‐Caribbean origin in the homozygous state, although their neutropenia status was not documented [34]. The two siblings reported here showed prolonged fasting tolerance of over 16 h, compared to the typical 2–4 h in most GSD1b patients [35], which likely explains why they remained undiagnosed for GSD1b.

This mild phenotype is most likely related to a possible residual transport activity of the mutant protein, as indicated in functional studies of recombinant G6PT‐Arg415Ter overexpressed in COS‐1 cells [36]. In that study, the mutant retained approximately 47% of the transport activity of wild‐type G6PT, whereas other mutants associated with more severe and classical GSD1b phenotypes completely lacked microsomal glucose‐6‐phosphate uptake. Notably, and as expected, shorter truncation mutants exhibited lower residual activity, whereas longer mutants retained higher activity. When the stop codon was introduced between residues 418 and 427, the resulting mutants displayed transport activity comparable to wild‐type G6PT, indicating that these truncated mutants can retain substantial transport function in this experimental system. This observation is consistent with the finding that the Arg423Ter mutant preserves transport activity but not ER retention [29, 31, 32].

Regarding the absence or very mild neutropenia in these siblings, the delayed diagnosis meant they were not prescribed the typical GSD1b diet rich in complex carbohydrates with a high glycaemic index. It was previously observed that GSD1b patients typically have 2‐3‐fold higher blood levels of 1,5‐AG compared with G6PC3‐deficient patients or healthy controls, a difference attributable to regular intake of complex carbohydrates [28, 31]. In fact, Table 1 shows that pre‐treatment 1,5‐AG concentrations in these two siblings were approximately 150 μM, compared with 350–400 μM reported in published GSD1b patients [37]. Since we have shown that 1,5‐AG6P concentrations in neutrophils are directly related to circulating 1,5‐AG levels [19, 30], it is likely that the combination of residual Arg415Ter activity and the patients' lower blood levels of 1,5‐AG prevented excessive 1,5‐AG6P accumulation in neutrophils, thereby explaining their absent or very mild neutropenia.

Both siblings had prolonged fasting tolerance of over 16 h compared with the typical 4 h in most GSD1b patients. They had no requirement for medical intervention for hypoglycaemia in infancy as typically seen as a presenting feature of GSD 1b. On the other hand, bone density was also noted to be lower in the younger sibling, in agreement with impairment of bone metabolism, as has previously been noted in poorly metabolic controlled patients with hepatic GSDs [38].

The late diagnosis of both siblings is surprising as by then they had completed several episodes of religious fasting without requiring medical attention. It is unclear if they experienced significant subclinical hypoglycaemia at these times. Retrospectively, the younger sibling was noted to be less active and more fatigable than his older sibling, suggesting potentially hypoglycaemia during these fasts.

Empagliflozin had a marked effect on many laboratory parameters for the younger sibling, including improved neutrophil count and reduced 1,5‐AG levels. Clinically, there were no episodes of infection, development of inflammatory bowel disease, or symptomatic hypoglycaemia since starting therapy, in agreement with prior experience of empagliflozin therapy in GSD1b [39]. None of the known side effects, such as genito‐urinary irritation and infection [24].

To our knowledge, these are the first reported patients with attenuated GSD1b presenting with hepatic and metabolic complications but without significant hypoglycaemia or neutropenia. The cases demonstrate the importance of considering a hepatic glycogen storage disorder as a cause of premature liver disease (hepatic steatosis or fibrosis) even in the absence of a significant history of hypoglycaemia.

## 5 Conclusions

GSD1b may be diagnosed in adulthood with chronic liver disease rather than hypoglycaemia. Differing phenotypes may be observed even in individuals with the same genotype.

## Author Contributions

G.L., K.M.S., M.V.C., A.W. – conceptualization, M.V.C., C.D., P.N. – data interpretation, G.L., M.V.C., K.M.S. – writing. All authors reviewed the last version of the manuscript.

## Funding

The authors have nothing to report.

## Ethics Statement

The authors have nothing to report.

## Consent

The written consent was obtained from both patients.

## Conflicts of Interest

The authors declare no conflicts of interest.

## Supporting information


**Table S1:** Case 1 laboratory results aged 33 years old.
**Table S2:** Calorific intake and nutrition profile for Case 1 aged 29–34 years old.
**Table S3:** Case 2 laboratory results including comparison between values before and after treatment with empagliflozin.
**Table S4:** DEXA scan results for Case 2 aged 28 years old.
**Table S5:** Dietary intake and nutrition profile for Case 2 aged 26–31 years old.
**Figure S1:** MRI Liver T2 weighted image in Case 1 aged 31 years old showing cirrhotic appearance and hepatic nodule.

## Data Availability

The data that support the findings of this study are available from the corresponding author upon reasonable request.
