# Supplementary material for: Clinical Outcomes and Management in Late Diagnosed Siblings Affected With Attenuated GSD Ib
Source: JIMD Rep. 2026 Feb 26;67(2):e70079. doi: 10.1002/jmd2.70079 (PMC12946512; doi:10.1002/jmd2.70079)
Supplement: Supplementary file 1 — Table S1: Case 1 laboratory results aged 33 years old. Table S2: Calorific intake and nutrition profile for Case 1 aged 29–34 years old. Table S3: Case 2 laboratory results including comparison between values before and after treatment with empagliflozin. Table S4: DEXA scan results for Case 2 aged 28 years old. Table S5: Dietary intake and nutrition profile for Case 2 aged 26–31 years old. Figure S1: MRI Liver T2 weighted image in Case 1 aged 31 years old showing cirrhotic appearance and hepatic nodule. [file JMD2-67-e70079-s001.docx]

Supplementary Materials


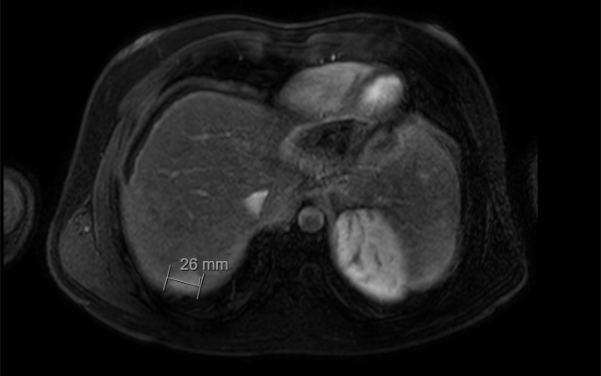


**Supplementary Figure 1.** **MRI Liver T2 weighted image in Case 1 aged 31 years old showing cirrhotic appearance and hepatic nodule.**

**Supplementary Table 1. Case 1 laboratory results aged 33 years old.**

| **Biochemistry** | Result | Reference Range | Median results |
| --- | --- | --- | --- |
| Urine ACR mg/mmol | <0.17 | <0.3 |  |
| Urine PCR mg/mmol | 4 | <25 |  |
| **Lipid profile (fasted)** |  |  |  |
| Total Cholesterol mmol/L | 1.5 | <5.0 | 1.8 |
| HDL Cholesterol mmol/L | 1.07 | >1.0 |  |
| Triglycerides mmol/L | 1.2 | <1.7 | 1.5 |
| HbA1c mmol/mol | 32 | 26 - 41 |  |
| Urate umol/L | 370 | 200 - 430 | 436 |
| Lactate mmol/L | 0.65 | 0.5 - 2.2 |  |
| Glucose mmol/L | 4.0 | 3.0 - 6.0 |  |
| ALT |  | 7-40 | 57 |
| **Haematology** | **Result range (2007 – 2011)** |  |  |
| Neutrophil count | 2.97 - 3.29 x 10^9/L | 1.5 - 8.0x10^9/L | 1.5 - 8.0x10^9/L |

**Supplementary Table 2~~.~~ Calorific intake and nutrition profile for Case 1 aged 29 – 34 years old. His height is 174cm.**

| Age (years) | Weight (kg) | BMI (kg/m^2^) | CGMS - % blood glucose 4 -10 mmol/L | CGMS % blood glucose 3.0 - 3.9 mmol/L | CGMS % blood glucose <3.0 mmol/L | Estimated average daily protein (g) | Estimated average daily energy intake (kcal) |
| --- | --- | --- | --- | --- | --- | --- | --- |
| 29 | 70.1 | 23.9 | 95.0 | 3.0 | 0 | 68.5 | 1800 |
| 31 | 68.6 | 23.4 | / | / | / | 70.0 | 1750 |
| 32 | 70.4 | 24.0 | / | / | / | 65.0 | 1850 |
| 33 | 69.8 | 23.8 | 85.0 | 14.0 | 0 | 68.0 | 1900 |
| 34 | 70.4 | 24.0 | 96.0 | 0 | 0 | 70.0 | 1850 |

0-no events of low glucose; /-CGM not done

**Supplementary Table 3.** **Case 2 laboratory results including comparison between values before and after treatment with empagliflozin*.***

| **Biochemistry** | Aged 25 (before empagliflozin) | Aged 28 (on empagliflozin) | Reference Range |
| --- | --- | --- | --- |
| Urine ACR (mg/mmol) | 0.79 | <0.3 | <0.3 |
| Urine PCR (mg/mmol) | 9 | <3 | <25 |
| **Lipid profile (fasted)** |  |  |  |
| Total cholesterol (mmol/L) | 7.3 | 3.9 | <5.0 |
| HDL cholesterol (mmol/L) | 1.51 | 1.72 | - |
| LDL cholesterol (mmol/L) | - | 0.9 | - |
| Triglycerides (mmol/L) | 6.7 | 2.9 | 0.4 - 1.5 |
| HbA1c (mmol/mol) | 32 | 34 | 26 - 41 |
| Urate (µmol/L) | 354 | 293 | 200 - 430 |
| Lactate (mmol/L) | 1.52 | 1.66 | 0.5 - 2.2 |
| Glucose (mmol/L) | 4.2 | 4.4 | 3.0 - 6.0 |
| **Haematology** |  |  |  |
| Neutrophil count (counts/L) | 1.0 - 1.86 x10^9 | 2.0 x10^9 | 1.5 - 8.0 x10^9 |
| Transferrin glycoforms | Transferrin glycoforms- normal pattern |  |  |

**Supplementary Table 4.** **DEXA scan results for Case 2 aged 28 years old.**

| Lumbar spine (L1-L4): | BMD 0.896 g/cmÂ², | Z-score -2.7 |
| --- | --- | --- |
| Left femur (Neck): | BMD 0.904 g/cmÂ², | Z-score -1.4 |
| Left femur (Total): | BMD 0.867 g/cmÂ², | Z-score -1.8 |

**Supplementary Table 5.** **Dietary intake and nutrition profile for Case 2 aged 26 –31 years old. His height is 169.5cm.**

| Age (years) | Weight (kg) | BMI (kg/m^2^) | CGMS- % blood glucose between 4 -10 mmol/L | CGMS % blood glucose between 3.0 and 3.9 mmol/L | CGMS % of blood glucose <3.0 mmol/L | Estimated average daily protein (g) | Estimated average daily energy intake (kcal) |
| --- | --- | --- | --- | --- | --- | --- | --- |
| 26 | 58.2 | 20.3 | 86.0 | 11.0 | 1.0 | 61.0 | 1700 |
| 27 | 60.4 | 21.1 | / | / | / | 70.0 | 1850 |
| 28 | 61.0 | 21.3 | / | / | / | 68.0 | 1850 |
| 29 | 61.0 | 21.3 | 75.0 | 25.0 | 0 | 60.0 | 1700 |
| 29 | 61.0 | 21.3 | 93 | 6.0 | 1.0 | 70.0 | 1850 |
| 31 | 62.2 | 21.7 | 96.0 | 3.0 | 1.0 | 70.0 | 1850 |
